# Quality in Context: The Role of Social Determinants of Health in Pediatric Quality Improvement

**DOI:** 10.1097/pq9.0000000000000036

**Published:** 2017-08-10

**Authors:** Deena J. Chisolm

**Affiliations:** From the Department of Pediatrics, The Ohio State University College of Medicine, Columbus, Ohio.

In the nearly 2 decades since the Institute of Medicine’s seminal publication of “Crossing the Quality Chasm,”^1^ health-care providers and systems have focused doggedly on maximizing the quality of care provided to patients in medical settings. Within the pediatric realm, significant improvements have been demonstrated. Yet, even with these improvements, many quality of care and health outcome goals have not been achieved and significant disparities by socio-economic status and racial/ethnic groups remain.^2,3^ Addressing these seemingly intractable challenges requires quality professionals to expand their focus beyond the health care system to the contexts in which children and families live their lives.

The term “social determinants of health” (SDH) refers to the social, economic, environmental, and demographic factors that influence health outcomes. These are “the conditions in which people are born, grow, work, live, and age, and the wider set of forces and systems shaping the conditions of daily life.”^4^ SDH range from resource deprivation (house instability, food instability), to environmental insults (air pollution, lead polluted water), to hardships and hassles of daily living (lack of transportation, child care). It is estimated that these factors are responsible for as much as 70% of overall health in a population with the remaining 30% attributable to health care and genetic factors.^5^ Even if we reach our clinical quality and safety goals, we cannot reach our population health goals without addressing the broader factors that influence health. Existing quality improvement (QI) frameworks have the flexibility to meet this challenge but, to maximize success, SDH concerns must be baked in not bolted on in every phase of project design and implementation.

## DEFINING THE PROBLEM

The first step in any QI effort is defining the problem. Although not every QI problem has an SDH component, many may. An SDH-informed approach to QI requires that the problem definition process reach beyond the clinical setting and ask “What contextual factors in the life of a child or family are barriers to our quality and outcomes goals?” Although factors like housing instability or food insecurity may be of concern across patient populations, other factors are diagnosis-specific, for example traffic-related air pollution for asthma patients. Understanding the key social drivers that matter for each population requires gathering information directly from patients and families in that population, using tools including SDH screeners in the clinic and focus groups of patients, families, and advocates in the community. In the pediatric population, this information gathering must explicitly consider factors that are important for the child and factors that are important for the parent/caregiver, recognizing that they may not be the same. For adolescents, that may even mean collecting SDH-related information independently from the parent. It is important to note though, that however SDH data are collected, it is imperative that nonjudgmental, culturally sensitive communication methods be used to place the emphasis on problem solving rather than patient blaming.^6,7^

## DESIGNATING THE DRIVERS

Once the problem is defined, QI teams are charged with developing a framework for action by determining the problem’s antecedents, sometimes referred to as “key drivers.”^8^ However, despite intentions to be exhaustive in the process of identifying drivers, SDH-related factors are often omitted from these frameworks, in part because some clinicians and administrators believe that social factors and social factor interventions are beyond the scope of health care system–based QI. It can be argued though that, at a minimum, QI teams should list SDH so that no one loses focus on the fact that they matter and they may influence the success of the proposed intervention. At best, QI teams should actually develop interventions for the SDH that are most amendable to change lead by the clinical system. In either case, SDH must not be kept hidden because they may explain why QI efforts fail or may point toward specific populations in which seemingly failed interventions may be successful.

## THINKING OUTSIDE THE WALLS

No one questions the inclusion of nurses, physicians, technicians, social workers, office staff, quality engineers, and administrators in QI teams. However, QI efforts have been somewhat reluctant to reach beyond the doors of the health care system. Although it may be understandable when some clinicians argue that they cannot be held accountable for what happens in the schools, it is less understandable when they deliberately do not bring the schools into the improvement conversation. Integration of SDH means going from multidisciplinary teams to multisectoral teams that are engaged throughout the process. QI programs that directly address SDH will need to engage representatives from housing, transportation, education, public safety, parks and recreation, and beyond. Such expanded stakeholder collaboratives generate new challenges because of separate mission priorities, funding streams, and sometimes languages, but it is the only way to address interlocking influences on health.

## DEVELOPING NEW MEASURES OF SUCCESS

Every QI professional has felt a sense of accomplishment when a run chart reaches its desired goal line and stays there. We know what QI success looks like and we celebrate it. However, as we bring new collaborators from new sectors to our quality table, we will have to develop new measures of success. This is the real move from health care QI to health improvement. New outcomes measured may include things like missed school days, parental employment rates, and high school graduation rates. New processes measured should help us learn best practices for involving stakeholders and addressing SDH. These are the types of successes that engender engagement and excitement beyond clinical conference rooms. It may be hard to go beyond the measures we know how to influence, but change is the very definition of QI, so it’s what we have to do.

## CONCLUSIONS

Current QI approaches are necessary but not sufficient for achieving ever increasing expectations for improving processes and outcomes, containing costs, and maximizing population health. As such, QI must engage with context. That means thinking beyond the patient to the family, the community, and the society. Many QI efforts have started along this path but there is—as we say in the QI world—significant opportunity for improvement.
